# Influence of Nutrient (NPK) Factors on Growth, and Pharmacodynamic Component Biosynthesis of *Atractylodes chinensis*: An Insight on Acetyl-CoA Carboxylase (ACC), 3-Hydroxy-3-Methylglutaryl-CoA Reductase (HMGR), and Farnesyl Pyrophosphate Synthase (FPPS) Signaling Responses

**DOI:** 10.3389/fpls.2022.799201

**Published:** 2022-03-18

**Authors:** Jin Sun, Haoming Luo, Yuxin Jiang, Lijuan Wang, Chunping Xiao, Lili Weng

**Affiliations:** School of Pharmaceutical Sciences, Changchun University of Chinese Medicine, Changchun, China

**Keywords:** *Atractylodes chinensis*, NPK fertilizers, polyacetylenes, sesquiterpenes, key enzymes, gene expression

## Abstract

In the planting of crops, especially medicinal plants, formula fertilization is important for improving the utilization rate of elements, soil quality, crop yield, and quality. Therefore, it is important to study targeted fertilizer application schemes for sustainable agricultural development and environmental protection. In this study, an L_9_(3^4^) orthogonal design was used to conduct a field experiment to study the effects of NPK combined application on the growth and pharmacodynamic component biosynthesis of *Atractylodes chinensis* (DC.) Koidz. Results showed that after applying a base fertilizer at the seedling stage (late May), topdressing at the vegetative stage (late June) and fruit stage (late August) was beneficial to the growth and development of *A. chinensis*. The high concentrations of phosphorus were conducive to the accumulation of yield and effective components, and the best harvest time was after late October. Principal component analysis (PCA) showed that the comprehensive score of T6 treatment was the highest, indicating that the optimal fertilization scheme for the high yield and high quality of *A. chinensis* was (N_2_P_3_K_1_): N 180, P_2_O_5_ 225, and K_2_O 105 kg⋅ha^–1^. A signaling response analysis showed that during the growth and development of *A. chinensis*, the T6 fertilization scheme had clear effects on the activity and gene expression of the key enzymes acetyl-CoA carboxylase (ACC) and farnesyl pyrophosphate synthase (FPPS). Under the T4 [(N_2_P_1_K_2_): N 180, P_2_O_5_ 75, and K_2_O 210 kg⋅ha^–1^] fertilization scheme, the activity and gene expression of the key enzyme 3-hydroxy-3-methylglutaryl-CoA reductase (HMGR) were higher. Moreover, ACC was closely related to the synthesis of the polyacetylene component atractylodin, and FPPS played an important regulatory role in the synthesis of sesquiterpene components atractylenolide II, β-eudesmol, and atractylon. In summary, the high phosphorus fertilization scheme T6 could notably increase the yield of *A. chinensis*, and promote the accumulation of polyacetylene and sesquiterpene volatile oils by increasing the expression of ACC and FPPS. Therefore, we postulate that the precise application of nutrients (NPK) plays a vital role in the yield formation and quality regulation of *A. chinensis*.

## Introduction

*Atractylodes chinensis* (DC.) Koidz., belongs to the Asteraceae family; it is mainly distributed in East Asia, where it is known as “Cangzhu” in China and “Sojutsu” in Japan (Toshinari et al., 2020; Wang L.Q. et al., 2020). The rhizomes of *A. chinensis* are used as medicine, mainly for the treatment of humoral imbalances and digestive diseases (Hossen et al., 2018). The medicinal history of *Atractylodis Rhizoma* can be traced back to the Han Dynasty (206BC-220AD) when the first Chinese Pharmacopeia, “Shen-nong-ben-cao-jing,” called it “Zhu.” Later, the medicinal materials were divided into *A. chinensis and Atractylodes lancea* (Thunb.) DC. (Cho et al., 2016).

The main components of the rhizomes of *A. chinensis* are volatile oils, such as polyacetylenes and sesquiterpenes, the polyacetylene component is mainly atractylodin. Acetyl-CoA carboxylase (ACC) is an important key enzyme in its biosynthetic pathway, and the expression of ACC is easily induced by external factors; e.g., jasmonic acid and fungi can increase or decrease its expression (Figueroa-Balderas et al., 2006; Romero-Munar et al., 2017). The components of sesquiterpenes include atractylon, β-eudesmol, and atractylenolide II, which are mainly synthesized in plants through the mevalonate (MVA) pathway, while 3-hydroxy-3-methylglutaryl-CoA reductase (HMGR) mainly catalyzes the formation of MVA (Aarthy et al., 2018). Farnesyl diphosphate (FPP) is the precursor of all sesquiterpenes, and farnesyl pyrophosphate synthase (FPPS) catalyzes the synthesis of FPP (Wen and Jia, 2021). Therefore, HMGR and FPPS are essential in the biosynthesis of sesquiterpenes (Zhou et al., 2017).

With the sharp increase in demand for medicinal drugs, the wild resources of *A. chinensis* have been exploited indiscriminately and led to a sharp decline in the reserves of wild resources of *A. chinensis* in North China and Northeast China. Therefore, a standardized cultivation protocol for *A. chinensis* is urgently needed. Studies have shown that the synthesis of effective substances in medicinal plants is affected by various soil factors, such as macro elements N, P, K, and trace elements, such as Fe, Zn, and Mn (Singh and Misra, 2000; Aruna et al., 2020; Shiponi and Bernstein, 2021). Soil content of NPK can significantly affect the plant growth and development and the accumulation of medicinal substances; the effect of a mixed application is better than that of a single application (Bernstein et al., 2019). At the same time, the application of organic fertilizer, inorganic fertilizer, and compound fertilizer can ensure the supply of soil nutrients and improve the soil that can greatly increase the yield and quality of crops per unit area (Ferreira et al., 2019; Al-Amri, 2021). However, different crops have different demand ratios for NPK, and the addition of a single fertilizer at too high or too low level will affect growth and development (Bai et al., 2022). Therefore, the study of the effects of NPK on the growth and development of *A. chinensis* and the formation of pharmacodynamic components is needed to deliver a standardized plan for planting. The clarification of the molecular mechanism of bioactive substance biosynthesis in *A. chinensis* is key to improve the quality of medicinal materials and solving the shortage of resources.

In summary, this study used field experiments and the L_9_(3^4^) orthogonal experiment design to reveal the changes in physiological and ecological indicators, such as growth indexes (plant height, stem and leaf fresh weight, fibrous root fresh weight, and rhizome fresh weight) and pharmacodynamic components (atractylenolide II, β-eudesmol, atractylodin, and atractylon) of *A. chinensis* under different NPK applications. At the same time, by measuring the activity and gene expression of the key biosynthetic enzymes ACC, HMGR, and FPPS, to explore the molecular regulatory mechanisms of the synthesis of polyacetylenes and sesquiterpenes in the rhizomes of *A. chinensis* under different NPK applications. We aimed to lay a theoretical foundation for further improving the yield and quality of *A. chinensis*, and provide a basis for the reasonable application of fertilizers at planting.

## Materials and Methods

### Experimental Site

The test site was located in Changnong *A. chinensis* planting base, Ankou Town, Liuhe County, Jilin Province, China (42°12′37.65″N, 125°36′4.94″E, and altitude 399 m). The experiment was conducted on private land with the permission of the landowner and did not involve endangered or protected species. The basic physical and chemical properties of the soil were: pH 5.65, organic matter content 51.73 g⋅kg^–1^, available nitrogen content 41.89 mg⋅kg^–1^, available phosphorus content 94.40 mg⋅kg^–1^, and available potassium content 143.54 mg⋅kg^–1^.

### Experimental Design and Field Management

The experiment used annual *A. chinensis* seedlings with similar growth, no damage on the surface, and no pests or diseases; they were purchased from Changnong Industrial Group Co., Ltd. (Jilin, China). The L_9_(3^4^) orthogonal fertilization scheme was adopted, with 3 factors of NPK at 4 levels. The specific fertilization amounts are shown in Table 1. A total of 10 different fertilization treatments (3 replicates) were set, with a total area of 150 m^2^ and a total of 30 plots. Each plot was 3 m^2^ (1.5 m × 2.0 m), and the row spacing was 15 cm × 20 cm. A 0.5 m wide walkway was set between the plots, and a 40 cm deep plastic film was buried on both sides of the walkway to block the water and nutrient flow. Transplantation took place in May 2019, except for fertilizer application, other treatments (such as, irrigation, weeding, and protection measures) were consistent with base management. In late May (seedling period), an application of base fertilizer with N: 60.0%, P: 100.0%, and K: 33.3% was applied. Topdressing was conducted in late June (vegetative period) and late August (fruit period), with N: 20.0% and K: 33.3%. Fertilizer formations were: urea (N, total nitrogen ≥ 46%), calcium superphosphate (P_2_O_5_, total phosphorus ≥ 46%), and potassium sulfate (K_2_O, total potassium ≥ 50%). The fertilization method involved uniform spraying on the soil surface of different treatment combinations of NPK dissolved in the same volume of water.

**TABLE 1:** Types and amounts of fertilization designed by L_9_(3^4^) orthogonal experiment.

Treatments	Groups	Application amount (kg⋅ha^–1^)		
		N	P_2_O_5_	K_2_O
T0	N_0_P_0_K_0_	0	0	0
T1	N_1_P_1_K_1_	90	75	105
T2	N_1_P_2_K_2_	90	150	210
T3	N_1_P_3_K_3_	90	225	315
T4	N_2_P_1_K_2_	180	75	210
T5	N_2_P_2_K_3_	180	150	315
T6	N_2_P_3_K_1_	180	225	105
T7	N_3_P_1_K_3_	270	75	315
T8	N_3_P_2_K_1_	270	150	105
T9	N_3_P_3_K_2_	270	225	210

### Sample Collection and Growth Indexes Determination

At 30, 60, 90, 120, and 150 days post-transplantation (dpt), 6 plants in each parallel plot were randomly sampled. After measuring the growth indexes (plant height, stem and leaf fresh weight, fibrous root fresh weight, and rhizome fresh weight), the non-medicinal parts were removed and cleaned. Some parts were stored in a refrigerator at −80°C, and the remaining parts were dried in shade for further use.

### Determination of Pharmacodynamic Components

The dried rhizomes of *A. chinensis* were ground into fine powder and passed through a 50 mesh (300 μm) sieve. Methanol (50 ml) was added to 300 mg of the accurately weighed sample. The solution was thoroughly vortexed for 1 min and sonicated (power 250 W and frequency 40 kHz) for 60 min at room temperature. The extract was left to cool, and then weighed. The lost weight was made up with methanol, the mixture was shaken well and filtered through 0.2 μm polytetrafluoroethylene (PTFE) membrane filters for further high-performance liquid chromatography (HPLC)–UV analysis.

**FIGURE 1:**
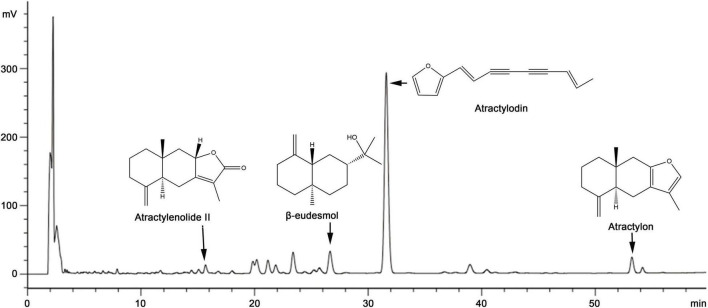
A typical high-performance liquid chromatography (HPLC)–UV chromatogram of several classical volatile oils presents in *Atractylodes chinensis*.

An Agilent Eclipse XDB-C18 reversed-phase chromatographic column (4.6 mm × 250 mm and 5 μm particle size) was used to determine the contents of atractylenolide II, β-eudesmol, atractylodin, and atractylon at a temperature of 32°C. The mobile phase was acetonitrile (A) and 0.2% phosphoric acid aqueous solution (B), and the flow rate was 1.0 ml/min. Gradient elution was performed as follows: 0–3 min, 55% solvent A; 3–20 min, 55%→60% solvent A; 20–35 min, 60%→65% solvent A; 35–55 min, 65%→85% solvent A; 55–60 min, 85%→95% solvent A; and holding at 100% solvent A for 10 min. UV detection wavelength was 0–28 min, 208 nm (atractylenolide II, β-eudesmol), 28–45 min, 340 nm (atractylodin), 45–60 min, 220 nm (atractylon) and the injection volume was 15 μl. As shown in Figure 1, the method was accurate, stable, and reproducible.

### Determination of Key Enzyme Activity

Enzyme activity was assayed according to the method of Kim et al. (2014), with slight modifications. The rhizome samples of *A. chinensis* were weighed (0.2 g), added to 1.8 ml precooled 0.01 mol⋅L^–1^ phosphate buffer saline (PBS) (pH 7.4, NaCl 8 g, Na_2_HPO_4_ 1.44 g, and KH_2_PO_4_ 0.24 g), and ground into a homogenate with liquid nitrogen. The slurry was centrifuged at 10,000 r⋅min^–1^ at 4°C for 20 min to remove the cell debris; then the supernatant was taken to determine the activities of ACC, HMGR, and FPPS using an enzyme-linked immunosorbent assay (ELISA) kit. The specific operation was as follows: take the supernatant (50 μl), add the corresponding enzyme labeled reagent (100 μl); leave at 37°C for 1 h, discard the solution, wash; color, using spectrophotometry at 450 nm wavelength absorbance; and calculate the activity of the sample.

### Quantitative RT-PCR Determination of Key Enzyme Genes

According to the instructions of the Takara plant total RNA extraction kit, total RNA was extracted from *A. chinensis* rhizomes. The concentration and purity of RNA were determined by a Multiskan Sky full wavelength enzyme labeling instrument, and it was reverse transcribed into cDNA. Using cDNA as the template and *EF-1*α as the internal reference gene (Sang et al., 2017), according to a 20 μl reaction system (SYBR^®^ Premix Ex Taq™ 10.4 μl, 10 μmol⋅L^–1^ Forward Primer 0.8 μl, 10 μmol⋅L^–1^ Reverse Primer 0.8 μl, cDNA template 2 μl, ddH_2_O 6 μl) and reaction program [95°C pre-denaturation for 30 s; 40 cycles (95°C denaturation for 5 s, and 64°C annealing for 1 min)]. The key enzyme genes *ACC*, *HMGR*, and *FPPS* were quantitatively analyzed for the different fertilizer combinations, and each sample had 3 biological replicates. Gene expression was calculated using the comparative threshold cycle (Ct) method. The levels of gene expression in all groups were expressed as a ratio to the control (seedling) group value. The primer sequences are listed in Table 2.

**TABLE 2:** The list of forward and reverse primers used in the gene expression study.

Genes	Forward	Reverse
*EF-1*α	ACCAACTGGGTT GACAACTGAAGT	AGCCTCGGTA AGGGCTTCAT
*ACC*	TCCAAGTTGG TGGCGGAAACAC	CGGTGACTGAA GCGGCATACAC
*HMGR*	AACCGCCACCAC TCACTCACATTCTTC	GGACGCCGT TGCTTCTGGAC
*FPPS*	TCTTGCGTGTG CCCTTGGTTG	TGACCTCTGCG TGTATGGGACTC

### Statistical Analysis

For each variable, SPSS 21.0, GraphPad prism 8.0, and Origin 2019 were used for the statistical analysis and graph plotting. All the data were analyzed by variance analysis. Student–Newman–Keuls post-special test was used to detect the multiple comparisons between treatments, *p* < 0.05. Principal component analysis (PCA) was used to analyze the selected pharmacodynamic components and physiological parameters of *A. chinensis*.

## Results

### Effects of Different NPK Combinations on the Growth Indexes of *Atractylodes chinensis*

Growth indexes reflect the vitality of plants, which is one important indicator for evaluating the adaptability of plants to soil environments. Changes in each index under different fertilization schemes are shown in Table 3. The results showed that: (i) the plant height of *A. chinensis* increased quickly 30–60 dpt. At 60-120 dpt, the plant height increased slowly. At 150 dpt, the average plant height under T3 scheme was the tallest (18.34 cm), which was 1.42-fold that of the control group. (ii) The variation trend of fresh weight of stem and leaf of *A. chinensis* at 30–120 dpt was similar to that of the plant height. Under different fertilization schemes, the stem and leaf fresh weight of T3 and T6 were relatively large. At 150 dpt, the plant entered the wilting stage, and fresh weight of the stem and leaf decreased. (iii) The fresh weight of fibrous roots of *A. chinensis* increased gradually during the growth period. At 30–90 dpt, there was little difference in the fresh weight of the fibrous roots under the different fertilizer combinations. At 150 dpt, the difference in the fibrous root fresh weight of each fertilization treatment was notable, and the T6 scheme was the largest, which was 1.41-fold of the control group. Similarly, (iv) rhizome fresh weight showed a gradual upward trend in the growth period. At 150 dpt, the fresh weight of rhizomes in each experimental group reached a maximum, and the average weight was 2.35–4.90 g. The fresh weight of rhizomes in the T6 scheme was the largest, and there was no notable difference between T3 and T6. It can be seen from the above that basal fertilizer application at the seedling stage (0 days), and topdressing at the vegetative growth stage (30 days) and fruit stage (90 days) were conducive to the growth and development of *A. chinensis*. Moreover, *A. chinensis* had good adaptability under the T3 and T6 fertilization schemes, and the plants showed good growth and vitality.

**TABLE 3:** The effects of different NPK combinations on the growth indexes of *Atractylodes chinensis.*

Growth indexes	Treatments	Transplanting time				
		30 d	60 d	90 d	120 d	150 d
Plant Height (cm)	T0	9.62 ± 2.19b	11.37 ± 1.03b	11.38 ± 1.15d	12.83 ± 1.00d	12.94 ± 0.74d
	T1	10.73 ± 2.25ab	15.72 ± 4.80a	15.30 ± 0.28abc	16.85 ± 1.70ab	16.80 ± 0.98ab
	T2	11.58 ± 1.05ab	15.60 ± 1.68a	15.77 ± 3.60abc	16.83 ± 2.63ab	17.20 ± 2.11ab
	T3	14.53 ± 2.61a	17.17 ± 2.84a	17.42 ± 1.10a	18.68 ± 0.70a	18.34 ± 1.24a
	T4	12.68 ± 0.76ab	14.75 ± 3.04ab	14.98 ± 2.50abc	15.98 ± 1.51bc	15.62 ± 1.49bc
	T5	11.97 ± 1.87ab	13.12 ± 2.31ab	12.88 ± 1.45cd	12.48 ± 1.35d	13.87 ± 1.90cd
	T6	13.70 ± 3.31a	16.47 ± 1.48a	16.35 ± 2.09abc	17.90 ± 0.98ab	17.62 ± 1.01ab
	T7	13.32 ± 3.03a	14.82 ± 0.52ab	14.85 ± 1.95abc	15.30 ± 1.92bc	15.52 ± 0.90bc
	T8	14.33 ± 1.49a	16.83 ± 1.56a	16.87 ± 2.52ab	17.88 ± 1.79ab	17.15 ± 1.10ab
	T9	9.53 ± 0.48b	12.85 ± 1.16ab	13.62 ± 0.98bcd	14.07 ± 1.22cd	13.75 ± 1.09cd

Stem and leaf fresh Weight (g)	T0	1.51 ± 0.22c	2.17 ± 0.31c	2.83 ± 0.16b	2.89 ± 0.24cd	1.71 ± 0.25e
	T1	1.83 ± 0.25bc	3.27 ± 0.50b	3.50 ± 0.42ab	3.94 ± 0.56ab	2.56 ± 0.40bc
	T2	2.46 ± 0.36abc	3.75 ± 0.57ab	3.84 ± 0.73ab	3.61 ± 1.02abcd	2.91 ± 0.39ab
	T3	2.90 ± 0.54a	4.33 ± 0.56a	3.98 ± 0.50a	4.39 ± 0.46a	3.19 ± 0.24a
	T4	2.55 ± 0.38ab	3.99 ± 0.39ab	3.67 ± 0.30ab	3.87 ± 0.50ab	2.94 ± 0.26ab
	T5	1.70 ± 0.45bc	2.49 ± 0.56c	2.87 ± 0.58b	2.83 ± 0.27d	1.81 ± 0.30de
	T6	2.43 ± 0.89abc	3.70 ± 0.26ab	3.53 ± 0.63ab	4.25 ± 0.39a	2.21 ± 0.39cd
	T7	2.30 ± 0.24abc	3.37 ± 0.36b	3.24 ± 0.89ab	3.33 ± 0.37bcd	1.99 ± 0.16de
	T8	2.45 ± 0.95abc	3.59 ± 0.52ab	3.80 ± 0.41ab	3.33 ± 0.33bcd	2.89 ± 0.22ab
	T9	2.45 ± 0.47abc	3.36 ± 0.53b	2.93 ± 0.61b	3.70 ± 0.46abc	2.24 ± 0.25cd

Fibrous root fresh Weight (g)	T0	1.36 ± 0.49a	1.94 ± 0.35b	2.84 ± 0.41ab	3.62 ± 0.35cd	4.46 ± 0.68bc
	T1	1.65 ± 0.72a	2.36 ± 0.47ab	3.59 ± 0.42ab	4.34 ± 0.19bc	4.94 ± 0.25abc
	T2	1.97 ± 0.49a	2.21 ± 0.76ab	2.84 ± 1.18ab	4.81 ± 0.38ab	5.51 ± 0.37ab
	T3	2.45 ± 0.41a	2.59 ± 0.18ab	4.00 ± 0.35a	5.10 ± 0.10a	5.80 ± 0.27ab
	T4	2.21 ± 0.24a	2.96 ± 0.61a	3.20 ± 0.32ab	4.02 ± 0.38bcd	5.70 ± 0.28ab
	T5	1.58 ± 0.54a	2.22 ± 0.53ab	2.46 ± 0.55b	3.35 ± 0.53d	4.13 ± 0.42c
	T6	2.49 ± 1.02a	2.63 ± 0.19ab	3.59 ± 0.75ab	5.47 ± 0.81a	6.29 ± 0.39a
	T7	2.39 ± 1.43a	2.95 ± 0.28a	3.20 ± 1.02ab	3.39 ± 0.44d	4.59 ± 0.30bc
	T8	2.07 ± 0.82a	2.17 ± 0.78ab	3.06 ± 0.40ab	4.28 ± 0.88bc	5.16 ± 2.22abc
	T9	1.99 ± 0.48a	2.81 ± 0.19ab	3.31 ± 0.31ab	4.16 ± 0.25bcd	5.88 ± 0.32ab

Rhizome fresh Weight (g)	T0	0.98 ± 0.43a	1.08 ± 0.23c	1.80 ± 0.16c	2.34 ± 0.17c	2.35 ± 0.12f
	T1	1.07 ± 0.14a	1.90 ± 0.24b	2.80 ± 0.81abc	3.83 ± 0.94ab	4.09 ± 0.52bc
	T2	1.22 ± 0.92a	1.66 ± 0.48bc	2.75 ± 1.11abc	3.69 ± 1.46ab	4.29 ± 0.22b
	T3	1.28 ± 0.78a	2.68 ± 0.72a	3.32 ± 0.21ab	4.36 ± 0.31a	4.88 ± 0.36a
	T4	1.51 ± 0.63a	1.95 ± 0.69b	2.77 ± 0.74abc	3.72 ± 0.44ab	4.55 ± 0.34ab
	T5	0.86 ± 0.17a	1.68 ± 0.73bc	2.29 ± 0.35bc	3.21 ± 0.59bc	3.22 ± 0.38de
	T6	1.68 ± 0.60a	2.92 ± 0.33a	3.53 ± 0.45a	4.56 ± 0.19a	4.90 ± 0.29a
	T7	1.55 ± 0.68a	1.69 ± 0.25bc	1.93 ± 0.29c	2.68 ± 0.35bc	2.90 ± 0.25e
	T8	1.51 ± 1.28a	1.83 ± 0.31bc	2.11 ± 0.93c	3.22 ± 0.35bc	3.36 ± 0.40de
	T9	1.81 ± 0.42a	2.03 ± 0.33b	2.56 ± 0.78abc	3.04 ± 0.50bc	3.68 ± 0.46cd

*(a) Initial growth index of A. chinensis seedlings: plant height 5.86 ± 1.08 cm; stem and leaf fresh weight 0.85 ± 0.31 g; fibrous root fresh weight 0.74 ± 0.17 g; rhizome fresh weight 0.72 ± 0.20 g (n = 6). (b) Different lowercase letters indicate that the effects of different NPK combinations on A. chinensis were significantly different in the same period (p < 0.05). (c) The composition of fertilization treatments: T0, N_0_P_0_K_0_; T1, N_1_P_1_K_1_; T2, N_1_P_2_K_2_; T3, N_1_P_3_K_3_; T4, N_2_P_1_K_2_; T5, N_2_P_2_K_3_; T6, N_2_P_3_K_1_; T7, N_3_P_1_K_3_; T8, N_3_P_2_K_1_; and T9, N_3_P_3_K_2_.*

### Effects of NPK Combined Application on the Concentration of Pharmacodynamic Components in *Atractylodes chinensis*

**FIGURE 2:**
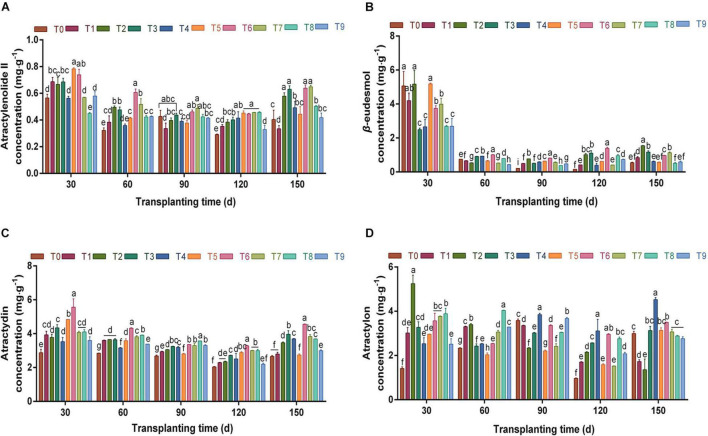
The effects of different NPK combinations on atractylenolide II **(A)**, β-eudesmol **(B)**, atractylodin **(C)**, and atractylon concentration **(D)** in rhizomes of *A. chinensis* (*n* = 3). (a) Initial effective component concentration of *A. chinensis* seedlings: atractylenolide II 0.2757 ± 0.0017 mg⋅g ^–1^; β-eudesmol 2.9488 ± 0.0958 mg⋅g^–1^; atractylodin 2.2449 ± 0.0132 mg⋅g^–1^; and atractylon 1.8074 ± 0.0421 mg⋅g^–1^. (b) Different lowercase letters indicate that the effects of different NPK combinations on *A. chinensis* were significantly different in the same period (*p* < 0.05). (c) The composition of fertilization treatments: T0, N_0_P_0_K_0_; T1, N_1_P_1_K_1_; T2, N_1_P_2_K_2_; T3, N_1_P_3_K_3_; T4, N_2_P_1_K_2_; T5, N_2_P_2_K_3_; T6, N_2_P_3_K_1_; T7, N_3_P_1_K_3_; T8, N_3_P_2_K_1_; and T9, N_3_P_3_K_2_.

The concentrations of the four main volatile oils in *A. chinensis* during the whole growth and development period were analyzed (Figure 2). The concentration of atractylenolide II in rhizomes changed in a “V”-shaped pattern during 30–150 dpt under the different NPK fertilization treatments (Figure 2A). The concentration of atractylenolide II showed a downward trend throughout 30–120 dpt, but the concentration was high under T6 scheme at each stage. At 150 dpt, the concentration of atractylenolide II increased, and the T7 scheme was the highest, which was 1.61-fold that of the control group. There were no significant differences between the T2, T3, T6, and T7 schemes (*p* > 0.05). Combined with the changes in rhizome fresh weight, it can be seen that the T6 fertilization scheme effectively promoted the synthesis and accumulation of atractylenolide II.

Under different NPK fertilization treatments, the concentration of β-eudesmol in the rhizome was the highest at 30 dpt, and was notably decreased between 60 and 150 days compared with 30 days (Figure 2B). At 120 dpt, the concentration of β-eudesmol was between 0.1588 and 1.3881 mg⋅g^–1^, and the β-eudesmol concentration was highest under the T6 fertilization scheme; furthermore, each fertilization group was significantly higher than that of the control group (*p* < 0.05). At 150 dpt, the concentration of β-eudesmol in T2 was the highest, which was increased by 51.92% compared with the previous period. Thus, exploring the variation law of yield and the content of medicinal components in different periods under each fertilization scheme, and clarifying the optimal harvest period of medicinal materials, are important measures for reducing agricultural production costs and improving economic benefits.

It can be seen from Figure 2C that during the whole growth and development period, the variation of atractylodin concentration in the rhizome of *A. chinensis* was similar to that of atractylenolide II. Under different fertilizer combinations in each period, the atractylodin concentration in the T6 scheme was significantly higher than that in the control group (*p* < 0.05). At 150 dpt, the concentration of atractylodin was 2.6588–4.5589 mg⋅g^–1^, and the T6 fertilization scheme was the highest, which was 1.71-fold that of the control group. It can be seen from the above that the T6 fertilization scheme significantly promoted the formation of sesquiterpene and polyacetylene volatile oils in the rhizomes of *A. chinensis*.

During the whole growth and development period, atractylon concentration in the rhizomes showed a “V”-shaped trend (Figure 2D). Between 90 and 150 dpt, the atractylon concentration under T4 was significantly higher than that under other fertilization treatments (*p* < 0.05). In the harvest period (150 days), the concentration of atractylon was 4.5252 mg⋅g^–1^, which was 1.51-fold that of the control group T0. It can be seen that a specific ratio of NPK can be targeted to improve the content of one or some components in crops, and provide a basis for promoting agricultural precision production through fertilizer application.

### Effects of NPK Combined Application on the Accumulation of Pharmacodynamic Components in *Atractylodes chinensis*

**FIGURE 3:**
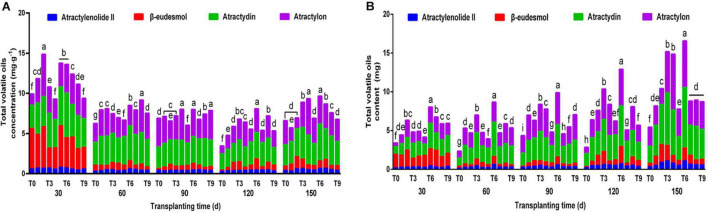
The effects of NPK combined application on total volatile oil concentration **(A)** and content **(B)** of *A. chinensis* (*n* = 3). Note: (a) The total concentration and content of volatile oil in *A. chinensis* seedlings were 7.2768 ± 0.1528 mg⋅g^–1^ and 1.8338 ± 0.0385 mg, respectively. (b) Different lowercase letters indicate that the effects of different NPK combinations on *A. chinensis* were significantly different in the same period (*p* < 0.05). (c) The composition of fertilization treatments: T0, N_0_P_0_K_0_; T1, N_1_P_1_K_1_; T2, N_1_P_2_K_2_; T3, N_1_P_3_K_3_; T4, N_2_P_1_K_2_; T5, N_2_P_2_K_3_; T6, N_2_P_3_K_1_; T7, N_3_P_1_K_3_; T8, N_3_P_2_K_1_; and T9, N_3_P_3_K_2_.

The proportion and total amount of effective components are important economic characteristics of Daodi herbs (Peng et al., 2021). Under different NPK combinations, the overall variation trend of the total concentrations of 4 volatile oils in rhizomes of *A. chinensis* was similar to that of single component concentration; however, the proportion of individual volatile oil concentration to the total concentration of all 4 volatile oils was different (Figure 3A). At 150 dpt, the total volatile oil concentration was 8.1594–16.5610 mg⋅g^–1^, and the concentration of the T6 scheme was the highest, which was increased by 46.36% compared with the control group. The accumulation of active ingredients determines the quality of medicinal materials. The contents of the 4 volatile oils were calculated by analyzing the concentrations of 4 effective components and the average rhizome weight. The biomass of *A. chinensis* increased with increased transplantation time (Table 3), and the total volatile oil content under different fertilization treatments showed an upward trend (Figure 3B). During the whole growth and development period, T3 and T6 schemes maintained high volatile oil content. At 150 dpt, the total volatile oil content of T3, T4, and T6 treatments increased most notably compared with the control group, and the total volatile oil content of T6 was the highest followed by T3 treatment. There was no significant difference between T4 and T3, indicating that different fertilizer ratios may also play a similar role in promotion, providing a basis for reducing fertilizer consumption and reducing agricultural production costs.

### Analysis of the Best Fertilization Plan for *Atractylodes chinensis* With Different NPK

**FIGURE 4:**
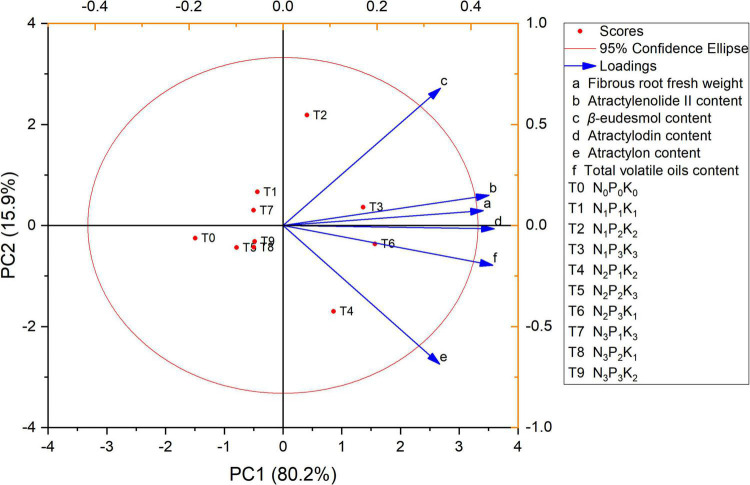
Multi-index principal component analysis (PCA) score map of *A. chinensis* under different NPK combined application.

Principal component analysis was used to comprehensively evaluate the yield and quality of *A. chinensis* under different NPK fertilization formulas during the harvest period (150 days). The component scores of the PCA are shown in Figure 4 for the case of PC1 and PC2 which contributed 96.1% of the variance. PC1 contributed up to 80.2% of total variance and PC2 contributed up to 15.9% of total variance. The differences among the treatments were mainly controlled by PC1. The treatments with an increase in NPK composition were found to be displaced toward the right along PC1 compared with the control treatment. The current research results demonstrated that under the soil fertility status represented by the experimental area, the optimal fertilization plan for the high yield and high quality of *A. chinensis* was N 180, P_2_O_5_ 225, and K_2_O 105 kg⋅ha^–1^. In other planting areas, this may need to be adjusted and optimized according to local soil conditions to achieve high-quality and high-yields.

### Effects of NPK Combined Application on Key Enzymes of *Atractylodes chinensis*

**FIGURE 5:**
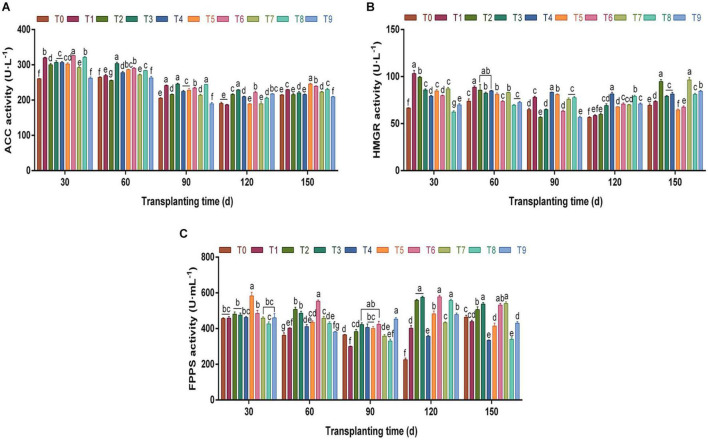
The effects of different NPK combinations on the activities of key enzymes acetyl-CoA carboxylase (ACC) **(A)**, 3-hydroxy-3-methylglutaryl-CoA reductase (HMGR) **(B)**, and farnesyl pyrophosphate synthase (FPPS) **(C)** in the rhizomes of *A. chinensis* (*n* = 3). (a) Initial key enzyme activities of *A. chinensis* seedlings: ACC 207.76 ± 1.85 U⋅L^–1^; HMGR 63.38 ± 1.19 U⋅L^–1^; and FPPS 326.67 ± 8.90 U⋅ml^–1^. (b) Different lowercase letters indicate that the effects of different NPK combinations on *A. chinensis* were significantly different in the same period (*p* < 0.05). (c) The composition of fertilization treatments: T0, N_0_P_0_K_0_; T1, N_1_P_1_K_1_; T2, N_1_P_2_K_2_; T3, N_1_P_3_K_3_; T4, N_2_P_1_K_2_; T5, N_2_P_2_K_3_; T6, N_2_P_3_K_1_; T7, N_3_P_1_K_3_; T8, N_3_P_2_K_1_; and T9, N_3_P_3_K_2_.

The key enzymes of volatile oil biosynthesis in *A. chinensis* were investigated (Figure 5). According to Figure 5A, compared with the control group T0, the ACC activity in rhizomes under different N, P, and K fertilization treatments changed significantly from 30 days to 150 dpt (*p* < 0.05), and the overall trend was “V”-shaped. The ACC activity decreased gradually from 30 days to 120 dpt. During this growth period, T3 and T6 schemes had a greater effect on the ACC activity; e.g., the ACC activity at 120 dpt was significantly higher than that of other fertilization groups (*p* < 0.05), which were 1.20- and 1.16-fold of the control group T0, respectively. At 150 dpt, ACC activity increased slightly compared with the previous period, and the range was 209.24–245.44 U⋅L^–1^.

Different from ACC, T3 and T6 with high phosphorus fertilization did not perform well in improving HMGR activity (Figure 5B). On the contrary, the T4 fertilization scheme greatly promoted HMGR activity in the middle and late growth stages of *A. chinensis*. For example, after 120 dpt, HMGR activity under T4 was significantly higher than that in other fertilization groups (*p* < 0.05), reaching 81.58 U⋅L^–1^, 1.44-fold that of the control group.

Under different NPK fertilization treatments, the overall changing trend of FPPS activity (Figure 5C) was similar to that of ACC. Between 60 and 150 dpt, the FPPS activities under T3 and T6 were significantly higher than those in the control group (*p* < 0.05). During the harvest period (150 days), the FPPS activities under T3 and T6 were 537.0833 U⋅ and 531.3889 U⋅ml^–1^, respectively, which were 15.88 and 14.65% higher than those in the control group. It can be seen from the above that the high phosphorus fertilization schemes T3 and T6 produced a great improvement in the activities of ACC and FPPS in the growth and development of *A. chinensis*, while T4 specifically improved the activity of HMGR.

### Effects of NPK Combined Application on Key Enzyme Genes of *Atractylodes chinensis*

**FIGURE 6:**
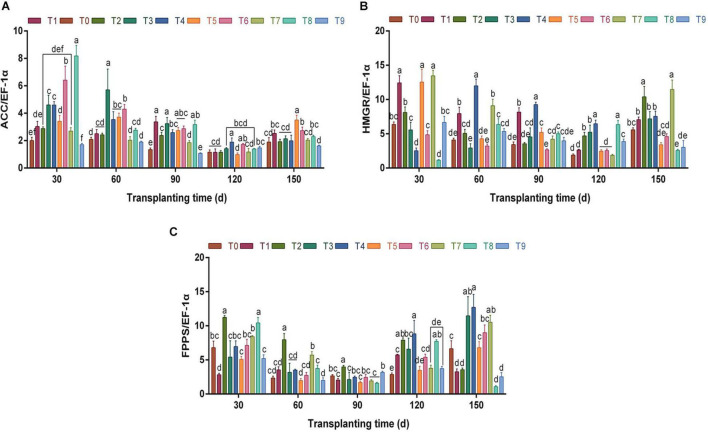
The effects of different NPK combinations on the relative expression levels of key enzyme genes *ACC* **(A)**, *HMGR* **(B)**, and *FPPS* **(C)** in the rhizomes of *A. chinensis* (*n* = 3). (a) The relative gene expression level is calculated based on the expression level of *A. chinensis* seedlings. (b) Different lowercase letters indicate that the effects of different NPK combinations on *A. chinensis* were significantly different in the same period (*p* < 0.05). (c) The composition of fertilization treatments: T0, N_0_P_0_K_0_; T1, N_1_P_1_K_1_; T2, N_1_P_2_K_2_; T3, N_1_P_3_K_3_; T4, N_2_P_1_K_2_; T5, N_2_P_2_K_3_; T6, N_2_P_3_K_1_; T7, N_3_P_1_K_3_; T8, N_3_P_2_K_1_; and T9, N_3_P_3_K_2_.

During growth and development, the relative expression of key enzyme gene *ACC* showed a “V”-shaped trend (Figure 6A). Under different fertilization schemes, the relative expression trend of the *ACC* gene was similar to that of enzyme activity, and the relative expression of *ACC* was high under T3 and T6 at each stage. Particularly, at 60 dpt, the schemes T3 and T6 were significantly higher than those of other fertilization groups (*p* < 0.05), which were 167.40 and 105.94% higher than those of the control group, respectively.

The relative expression of *HMGR* was similar to that of *ACC* (Figure 6B). At 30–120 dpt, the relative expression levels of *HMGR* showed a downward trend as a whole, and the expression levels under T4 were the highest between 60 and 120 dpt, which were 2.93-, 2.70-, and 3.43-fold that of the control group, respectively. At 150 dpt, the relative expression level of *HMGR* showed an upward trend compared with the previous period, and the relative expression level was between 2.57 and 11.46.

Different from the key enzyme genes *ACC* and *HMGR*, the relative expression level of *FPPS* (Figure 6C) was the lowest at 90 dpt. At this time, with the exception of the T2 scheme, there were no significant differences between other fertilization treatments and the control group (*p* > 0.05). Between 90 and 150 dpt, the relative expression of *FPPS* showed an overall upward trend. At 150 dpt, the relative expression of *FPPS* in T3, T4, T6, and T7 was significantly higher than that in the control group (*p* < 0.05). In summary, the changes of gene expression levels of the 3 key enzymes were similar to enzyme activities, which proved that precise fertilizer application could promote the synthesis and accumulation of metabolites by regulating the expression of enzymes and genes.

### Correlation Analysis of Pharmacodynamic Components and Key Enzyme Activity and Gene Expression of *Atractylodes chinensis* Under Different NPK Combined Applications

**FIGURE 7:**
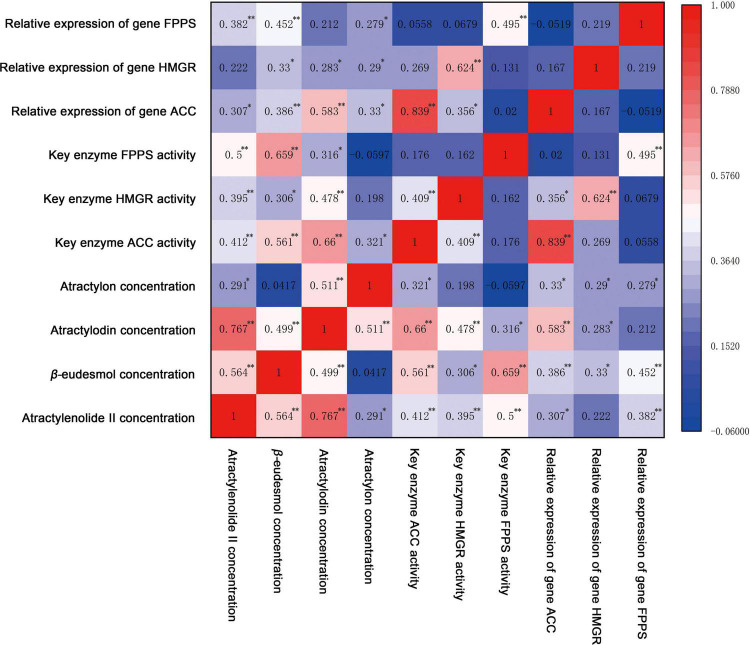
The correlation analysis of pharmacodynamic components with key enzyme activity and gene relative expression of *A. chinensis* under different NPK combination applications. * indicates significant correlation at *p* < 0.05 (bilateral), ** indicates significant correlation at *p* < 0.01 (bilateral).

Addressing the changes of pharmacodynamic components, key enzyme activities, and gene expression levels during the growth and development of *A. chinensis*, Spearman’s correlation analysis was performed on the concentrations of atractylenolide II, β-eudesmol, atractylodin, and atractylon, the activities of the key enzymes ACC, HMGR, FPPS, and the relative expression of key enzyme genes *ACC, HMGR*, and *FPPS*. The results are shown in Figure 7.

The correlation results (Figure 7) showed that there was a highly significant positive correlation between the concentration of polyacetylene component atractylodin and sesquiterpene components atractylenolide II, β-eudesmol, and atractylon (*p* < 0.01). The correlation between atractylodin and atractylenolide II was the highest (0.767), and the correlation between atractylodin and β-eudesmol and atractylon was slightly lower (0.499, 0.511). At the same time, atractylenolide II was significantly positively correlated with β-eudesmol (*p* < 0.01) and atractylone (*p* < 0.05). Among the activities of key enzymes ACC, HMGR, and FPPS, there was no correlation except that ACC was highly positively correlated with HMGR (0.409). Similarly, there was no correlation between the relative expression of key enzyme genes *ACC*, *HMGR*, and *FPPS*, indicating that the expression of 3 genes was relatively independent. However, there was a highly significant positive correlation between the corresponding genes and enzymes (*p* < 0.01). Among them, ACC activity had the highest correlation with the relative expression of the *ACC* gene (0.839), while HMGR and FPPS activities had a slightly lower correlation with the relative expression of the corresponding genes (0.624, 0.495).

The correlation analysis showed that atractylodin was positively correlated with the activities of key enzymes ACC, HMGR, and FPPS, and the relative expression of key enzyme genes *ACC* and *HMGR*. Among them, atractylodin was significantly positively correlated with the ACC enzyme and gene in the polyacetylene biosynthesis pathway, and the correlation coefficient was large (0.660, 0.583). Meanwhile the correlation coefficient with enzymes HMGR and FPPS was small (0.478, 0.316), indicating that polyacetylene atractylodin was mainly regulated by ACC and its genes, and was also affected by HMGR and FPPS. Atractylenolide II was significantly positively correlated with the activities of ACC, HMGR, and FPPS (*p* < 0.01); the correlation coefficients were 0.412, 0.395, and 0.500, indicating that the biosynthesis of atractylenolide II was mainly regulated by FPPS and ACC. β-eudesmol was positively correlated with the 3 key enzymes and genes to varying degrees, and was extremely significantly positively correlated with the activities of FPPS, ACC, and gene expression (*p* < 0.01). Among them, the correlation coefficient of FPPS was the largest (0.659), indicating that the biosynthesis of β-eudesmol in rhizomes was mainly regulated by FPPS. Atractylon was significantly correlated with the expression of key enzyme genes *ACC*, *HMGR*, and *FPPS* (*p* < 0.05), and had a similar correlation coefficient, indicating that the synthesis of atractylon in rhizomes was affected by 3 genes. In summary, the synthesis of volatile oil in the rhizomes of *A. chinensis* is a complex process involving multiple enzymes and genes. ACC and FPPS play important regulatory roles in the biosynthesis of polyacetylenes and sesquiterpenes, respectively.

## Discussion

### Insights Into the Impact of NPK on Growth Indexes

Formula fertilization is an advanced agricultural technology for the scientific application of large amounts of the elements and trace elements according to the laws of crop fertilizer demand, soil fertilizer supply performance, and fertilizer effect (Mary Isabella Sonali et al., 2022). This technology can not only improve the yield of cash crops, but also improve their quality, which greatly promotes research regarding plant-soil fertilizer systems (Habibur et al., 2021). This study clearly indicated that the availability of nutrients (NPK) had a significant impact on *A. chinensis* productivity. Initially, we found that the growth of *A. chinensis* plant height and stem leaf fresh weight showed better adaptability and responsiveness to the T3 scheme, which highlighted that the higher levels of phosphorus and potassium content in soil could make the plant stems robust and leaves lush. Phosphorus is involved in energy metabolism and photosynthesis during plant growth (Yan et al., 2021), and potassium plays an important role in carbohydrate and protein metabolism (Hassanein et al., 2021), indicating that a reasonable proportion of nutrition (NPK) factors directly promote the absorption and assimilation of plants, thereby affecting their growth and development (Yildirim et al., 2011). Subsequently, we analyzed the fresh weight of fibrous roots and the rhizomes of *A. chinensis*, because these two important attributes directly determine the yield of crops. The results showed that under high phosphorus fertilization schemes (T3, T6) both were significantly greater than in other treatment groups. Therefore, we speculated that the higher levels of soil phosphorus would significantly promote the root development of *A. chinensis*. Excitingly, other researchers have observed similar effects of phosphorus content in soil on crop roots, which provided an important basis for the study of an *A. chinensis* fertilization formula (Pelá et al., 2018).

### Insights Into the Impact of NPK on Pharmacodynamic Components

The scientific and precise application of NPK is conducive for increasing crop yields (Jabborova et al., 2021). The planting of Chinese medicinal materials should not only consider yield, but also pay attention to the quality (Ostadi et al., 2020). These 2 jointly determine the cost and benefit of subsequent processing, which is of great importance in production. In this study, we used different ratios of NPK to regulate soil nutrient composition (Table 1). When plants can obtain plentiful essential nutrients, the production of secondary metabolites will be greatly promoted (Wang J. et al., 2020). However, the effects of different NPK ratios on metabolic pathways are different. We found that the metabolic pathways of polyacetylenes and sesquiterpenes showed a good response to the T6 fertilization treatment, which was mainly manifested by a notable increase in the concentrations of atractylodin, β-eudesmol, and atractylenolide II. Interestingly, T4 fertilization treatment could specifically increase the concentration of the sesquiterpene atractylon, which stimulated ideas for the development of precision agriculture. The comprehensive analysis of the 4 effective components (Figure 3) showed that T6 fertilization notably increased the accumulation of total volatile oil in *A. chinensis*, and the harvest content was the highest after late October. Therefore, the current research results demonstrated that the greater application of phosphorus fertilizer can allow *A. chinensis* to achieve the higher yields of volatile oil. Similarly, Meysam et al. (2017) found that the application of phosphate fertilizer could increase the total volatile oil production of *German Chamomile*. We speculated that this may be closely related to the participation of phosphorus in the composition of key molecules, such as nucleic acids and ATP (Campos et al., 2018).

In addition, a single analysis may not be sufficient to determine the relationship between physiological performance and soil nutrient factors in soil-plant systems, which should be combined with other analytical procedures. The biological laws of soil nutrient factors on plants need to be further explored and analyzed. PCA uses variable rotation to reduce variable dimensions and create one or more new variables (Sun et al., 2012). In this study, PCA was used to evaluate the correlation between nutritional factors and physiological characteristics (Figure 4). PCA study showed that the T6 (N_2_P_3_K_1_) fertilization scheme had the best effect; this was the optimal and most favorable fertilizer composition for improving the yield and quality of *A. chinensis*. It should be noted that optimizing NPK composition to achieve efficient and sustainable agricultural production seems to involve complex processes (Roy et al., 2020a,b, 2021a,b), which would totally depend on the harmonious behavior of plant-soil systems (Wang J. et al., 2020). However, for the long-term effect of NPK fertilizer, such optimization is necessary. The current research offers new insights for sustainable agricultural research and crop fertilization formulation, suggesting that accurately adding NPK in certain proportions or compositions according to plant characteristics, will be reliable and cost-effective.

### NPK, Pharmacodynamic Components, Key Enzymes, and Genes Interactions

The accumulation mode of secondary metabolites in plants is multicomponent and multi-active (Chen et al., 2007), and the purpose of regulating plant secondary metabolism is to promote the accumulation of some active compounds (Zhou et al., 2018). However, plant secondary metabolites are regulated by many enzymes and genes owing to their diversity and complex synthesis mechanisms (Pickett, 2014). Therefore, the in-depth exploration of metabolic pathways is key to clarifying the molecular mechanism of the biosynthesis of medicinal substances, improving the quality of medicinal materials, and solving the shortage of medicinal plant resources (Zhang et al., 2021). This study found that fertilizer application can induce changes in enzymes and gene levels in plants, promote the accumulation of secondary metabolites, and thus enhance plant growth and competitive advantage in the natural environment (Krausfeldt et al., 2019). Among them, the promotion effects of T3 and T6 fertilization schemes on the activities and gene expression of key enzymes ACC and FPPS were notable. The T4 fertilization treatment was the superior scheme for HMGR activity and gene overexpression. This well corresponds to the accumulation level of total volatile oil in the rhizomes of *A. chinensis* (Figure 3), fully indicating that precision fertilizer application can effectively regulate the expression of key enzymes and genes in synthesis pathways, and thus effectively promote the accumulation of metabolites (Ali et al., 2021). In addition, we found a significant correlation between key enzyme activity and gene expression and the concentration of active ingredients (Figure 7). These results clearly show that the upregulation of ACC enzymes and genes are key factors in the synthesis of atractylodin, a polyacetylene component. The expression of FPPS enzyme and gene is essential for the synthesis of sesquiterpene components atractylenolide II, β-eudesmol, and atractylon. HMGR enzymes and genes regulate the synthesis of β-eudesmol, atractylodin, and atractylon to a certain extent. Therefore, we speculated that the high phosphorus fertilization scheme T6 could not only increase the accumulation of medicinal parts of *A. chinensis* through primary metabolism, but also further activate secondary metabolism to promote the synthesis and accumulation of volatile oil components by increasing the expression of ACC and FPPS, which is similar to the findings of a new study on medical cannabis (Shiponi and Bernstein, 2021).

In summary, we believe that exploring the effects of nutrition (NPK) factors on the traits and qualities of different medicinal plants and developing specific and targeted fertilizer application schemes are of great import for promoting agricultural precision production, reducing agricultural production costs, and improving the quality of medicinal materials. At the same time, we recognize that the interaction between fertilizer application and environmental factors (light, temperature, and water conditions) should be strengthened in a future study.

## Conclusion

The purpose of this study was to investigate the effects of nutrients (NPK) on the growth indexes (plant height, stem and leaf fresh weight, fibrous root fresh weight, and rhizome fresh weight), pharmacodynamic components (atractylenolide II, β-eudesmol, atractylodin, and atractylon), key enzymes (ACC, HMGR, and FPPS), and the genes of *A. chinensis*. Under different NPK combinations, the T6 (N_2_P_3_K_1_) scheme notably promoted the yield and volatile oil content of *A. chinensis*. Therefore, we believe that the process of planting *A. chinensis* involves, “heavy application of P fertilizer, and appropriate application of N and K fertilizer.” In addition, we noticed that the synthesis of polyacetylene atractylodin was closely related to ACC; furthermore, FPPS had an important regulatory effect on the synthesis of sesquiterpenes atractylenolide II, β-eudesmol, and atractylon. At the same time, the T6 fertilization program notably improved the key enzymes ACC and FPPS activity and gene expression. In summary, reasonable NPK administration can both effectively promote the growth of the medicinal parts of *A. chinensis*, and also promote the accumulation of medicinal ingredients by regulating the expression of key enzymes and genes in the synthesis pathway.

## Data Availability Statement

The datasets presented in this study can be found in online repositories. The names of the repository/repositories and accession number(s) can be found in the article/supplementary material.

## Author Contributions

JS: data curation, formal analysis, software, and writing-original draft. HL: investigation and methodology. YJ: sample collection and data curation. LjW: methodology. CX: conceptualization, project administration, funding acquisition, and writing-review & editing. LlW: conceptualization, funding acquisition, and supervision. All authors contributed to the article and approved the submitted version.

## Conflict of Interest

The authors declare that the research was conducted in the absence of any commercial or financial relationships that could be construed as a potential conflict of interest.

## Publisher’s Note

All claims expressed in this article are solely those of the authors and do not necessarily represent those of their affiliated organizations, or those of the publisher, the editors and the reviewers. Any product that may be evaluated in this article, or claim that may be made by its manufacturer, is not guaranteed or endorsed by the publisher.
